# Equity for Open-Access Journal Publishing

**DOI:** 10.1371/journal.pbio.1000165

**Published:** 2009-08-04

**Authors:** Stuart M. Shieber

**Affiliations:** School of Engineering and Applied Sciences, Harvard University, Cambridge, Massachusetts, United States of America

## Abstract

Open-access journals, which provide access to their scholarly articles freely and without limitations, are at a systematic disadvantage relative to traditional closed-access journal publishing and its subscription-based business model. A simple, cost-effective remedy to this inequity could put open-access publishing on a path to become a sustainable, efficient system.

Scholars write articles to be read—the more access to their articles the better—so one might think that the open-access approach to publishing, in which articles are freely available online to all without interposition of an access fee, would be an attractive competitor to traditional subscription-based journal publishing.

But open-access journal publishing is currently at a systematic disadvantage relative to the traditional model.

I propose a simple, cost-effective remedy to this inequity that would put open-access publishing on a path to become a sustainable, efficient system, allowing the two journal publishing systems to compete on a more level playing field. The issue is important, first, because academic institutions shouldn't perpetuate barriers to an open-access business model on principle and, second, because the subscription-fee business model has manifested systemic dysfunctionalities in practice. After describing the problem with the subscription-fee model, I turn to the proposal for providing equity for open-access journal publishing—the open-access compact.

Economists use the term “moral hazard” for the phenomenon of overconsumption of a good by a consumer who is insulated from the good's cost. The concept is frequently associated with the health care industry, in which insurance can lead to moral hazard in an insured's behavior of overconsumption of medical care. In fact, there is empirical evidence for moral hazard in medicine, and the hyperinflation of medical costs follows in part from this problem. Co-payments and deductibles, by passing on a portion of costs to the consumer, serve as tools to mitigate this moral hazard in medicine.

Moral hazard exists in scholarly publishing as well. The “consumers” of scholarly articles (the readers, typically faculty, students, and researchers at universities and other research institutions) are insulated from the cost of reading, that is, from the subscription fees paid by the institutions' research libraries. The expected result—inelasticity of demand and hyperinflation—can be amply seen in the statistics of serials costs paid by research libraries [1]. As subscription fees hyperinflate, libraries with budgets that at best merely match inflation must inevitably drop subscriptions, reducing access to the scholarly literature. The problem has been dramatically exacerbated by the current economic downturn. Some research institutions, including my own, are beginning to entertain wholesale elimination of subscription access to entire groups of serials, as library budgets take large cuts. Such elimination of access is bad for the scholarly enterprise, and the threat of unsustainability of journals is especially worrisome given the invaluable services that they provide to scholars: logistical management of the peer review process, production services such as copyediting and typesetting, distribution and preservation, and filtering and imprimatur based on a journal's “brand.”

But unlike access to medical care, where technological advances have dramatically increased the cost of access to state-of-the-art care (think MRI), access to scholarly articles has been reduced to essentially zero marginal cost, thanks to digital network technology (think hyperlink). In a world where the first-copy cost of publishing an article is essentially the entire cost, a business model for publishing that charges per article for article-processing services (the very services listed above) makes a lot of sense. It would in theory enable free and open access to articles while providing revenue to fund the important services that journals provide. The Public Library of Science journals are, of course, the flagship examples of journals using this business model. But there are several thousand other so-called open-access journals operating in all areas of scholarship. Although by definition they do not charge an access fee paid by or on behalf of a reader, they can still acquire revenue by charging an article processing fee paid by or on behalf of the author.

In fact, only a minority of the extant open-access journals actually charge processing fees, as first confirmed by the Kaufman-Wills Group [2] and reconfirmed in various ways subsequently. Currently, fewer than 25% of the open-access journals in the Directory of Open Access Journals are listed as charging a publication fee, the remainder relying on other sources of direct or in-kind support. (Perhaps surprisingly, more than half of the subscription-based journals charge processing fees of one sort or another [2].) Nonetheless, processing fees are likely to be an important revenue model for open-access journals, as they scale beyond the tiny fraction of overall journals that they currently constitute; processing fees are the only revenue source that inherently scales directly with the publishing services provided by a journal. The importance of the processing-fee model can be seen in the fact that of the open-access journals of sufficient standing to have an Institute for Scientific Information (ISI) impact factor, the proportion charging processing fees rises above 50%.

Over the past several decades, a workable infrastructure has developed to handle the subscription-based mechanism for scholarly journals—publishers to manage logistics and production, subscription agents to handle order processing, library budgets to pay for the subscriptions, overhead from grants to fund those library budgets, and so forth. Unfortunately, there is no such infrastructure to support the processing-fee model. Imagine you are a publisher of a subscription-fee journal, a forward-thinking publisher who sees the benefit to scholarship or at least the inevitability of the open-access processing-fee model. You would like to convert one of your journals to an open-access model. However, you realize that, were you to take this bold step, yours would be the first journal in its field to charge processing fees. Prospective authors would suddenly be faced with the prospect of paying, say, US$1,500 to publish their articles in your journal, as compared to paying nothing for your competitors' journals. Even though the primary motivation for authors is gaining the journal's imprimatur and the quality of that imprimatur for your journal hasn't changed (yet), the US$1,500 may be perceived as a steep price for the imprimatur advantage of your journal over your competitors'. By changing the business model for the journal, you risk abandonment of your journal by authors, undercutting its quality and its nascent revenue source. It might even be considered a derogation of your fiduciary duty to your shareholders to make the change in the face of such a high risk. Even a forward-thinking publisher is unlikely to take such a risk.

The problem is, of course, that the US$1,500 article revenue to the journal that is provided by the processing fee under the processing-fee model is hidden in subscription charges in the subscription-fee model, and these are typically paid not by the authors, even in their role as readers of the journals, but on their behalf by subscribing research institutions, typically university research libraries. The authors don't see these charges; hence, they don't enter their economic calculus. Yet authors are now expected to pay these charges under the open-access processing-fee model. It is no wonder they might be expected to submit preferentially to closed-access journals. Indeed, it is a wonder that authors, even now, are willing to submit to processing-fee-charging open-access journals. It is a testament to the power of journal imprimatur over immediate financial interest that authors are willing to submit their best work to such journals.

In summary, publishers see an unlevel playing field in choosing between the business models for their journals exactly because authors see an unlevel playing field in choosing between journals using the business models. To mitigate this problem—to place open-access processing-fee journals on a more equal competitive footing with subscription-fee journals—requires those underwriting the publisher's services for subscription-fee journals to commit to a simple “compact” guaranteeing their willingness to underwrite them for processing-fee journals as well.

The crucial underwriters are universities and funding agencies. Universities underwrite closed-access journals through the institutional subscriptions that they purchase. Funding agencies do so through the overhead charges that they provide to grantee institutions, a sizable (and specifically negotiated) fraction of which is applied to support of the libraries and thereby subscription fees again. Since both universities and funding agencies are (directly or indirectly) underwriting journal subscriptions, both should be involved in underwriting article-processing fees for open-access journals as well.

The crucial property of the proposed compact is that the funds disbursed must be nonfungible, that is, applicable only to open-access processing fees. The ability of authors to trade off the funds against other uses for the money (purchasing lab supplies or equipment, funding research assistants, and so forth) would provide a disincentive to use the funds to enable publishing in an open-access journal; no progress would be made on the underlying equity issue. The funds shouldn't, for instance, be folded into a general research fund. The nonfungibility property is crucial.

Funding agencies would ideally implement the compact by providing incremental funding for reasonable processing fees for articles in open-access journals, describing the results of research funded by their grants. By “incremental,” I mean that the funds would not come from the grant budget itself, but through a separate disbursement process whereby a grantee could request additional funds to pay open-access processing fees for articles based on grant-funded research. (The funding should be incremental primarily to guarantee nonfungibility and secondarily because those charges may reasonably be incurred some time after the grant period ends.) Wellcome Trust has pioneered exactly such a procedure for its grantees.

But not all research is grant-funded. Universities would commit on behalf of their authors to underwrite reasonable processing fees for articles in open-access journals for which funds are not otherwise available (in particular, for research not funded by grants).

There are many issues of implementation that would need to be addressed for a university or funding agency to turn the compact into a working program. Such issues as whose articles and which journals would be eligible, what constitutes a reasonable processing fee, what limitations on funds would be instituted and how they would be allocated, and so forth would all need specification. To a great extent, the details are incidental so long as they match the underlying goal of providing a sustainable mechanism for supporting open-access journals. Each university and funding agency will want to instantiate the compact as it sees best given its situation. It may nonetheless be useful to present some of my own opinions on aspects of implementation of such a compact, which I do below, keeping in mind that others may choose differently.

To be eligible for reimbursement, the venue of publication would have to be a “pure” open-access journal, that is, a journal that does not charge readers or their institutions for access to any of the peer-reviewed articles that it publishes. This does not mean that the journal cannot sell subscriptions to print issues of the journal. Some journals with substantial non-peer-reviewed content (in the *Science* and *New England Journal of Medicine* mold) might even charge subscription fees for online access to the full range of published material. It is only those portions of the journal that, in the language of the Budapest Open Access Initiative, “scholars give to the world without expectation of payment” that would be required to be available open access.

Journals with a hybrid open-access model or a delayed open-access model would not be eligible. Hybrid and delayed open-access journals already receive revenue through subscription charges, even for the articles that they make freely available. Publishers of hybrid journals sometimes maintain that they will prorate their subscription prices based on the proportion of articles not available open access. For instance, Springer says of its “Open Choice” hybrid program, “Springer plans to continue to evaluate its journal subscription prices on a yearly basis, based on a number of factors, including the amount of subscription-model content being published.” However, it is generally impossible to verify such claims given the price discrimination that publishers engage in. More importantly, the proration is not specific to the institution, so that any pricing benefit is shared among (and therefore diluted by) all subscribers. Even an institution that covered hybrid fees for all articles emanating from it would, at best, see a tiny compensating reduction in subscription fee. Consequently, the hybrid approach is subject to a “tragedy of the commons”; if all institutions participated fully, all would see subscription fees disappear, but no single institution sees any observable direct benefit by participation. It seems inappropriate to support a transitional business model with such an inherent flaw in its design.

In addition, eligible journals would be required to have a policy to substantially waive fees in case of economic hardship. The fee-waiver policy would not, of course, apply to compact-institution authors, but would ensure that compact funds are not supporting journals that may disenfranchise researchers with limited means. Such fee waivers are, in any case, quite common for open-access journals.

One might imagine that with funding agencies, universities, and other research institutions paying processing fees on behalf of their constituencies, a new moral hazard would be engendered; since authors would now not have to pay the processing fee, they would overconsume in a price-blind fashion, and processing fees would hyperinflate just as subscription fees have. The situation is not parallel, however, because the processing-fee approach intrinsically involves participation of the author. The simplest prescription to mitigate hyperinflation is to take advantage of the underlying principle of the compact to underwrite reasonable open-access fees. Fees above a certain level might be deemed unreasonable, leading to a cap per article on reimbursement of fees.

An even better alternative is to place the cap on the funding made available to an author overall. Authors would have to trade off whether using a certain amount of their limited allocation of funds for a given journal was appropriate in relation to the services and imprimatur that the journal provides, thereby reintroducing exactly the economic tradeoff that is missing from the current system. A funder could cap processing-fee expenditures as a fixed small percentage of the grant amount. A university could cap processing-fee expenditures at a fixed annual rate per author. The caps could be designed to be sufficient to cover standard costs of processing fees for authors who publish at a typical level, but would still serve as a limit that would force authors to trade off the cost of a publication against the services (especially the imprimatur) that a journal provides, in exactly the way that we would want in order to generate price pressure on publisher processing fees. In essence, the caps would act as inverse deductibles still allowing the economic signal to pass through to authors. In this approach, decisions about what is a reasonable fee are delegated to authors who choose on the basis of a market mechanism; the institution needn't stipulate reasonableness a priori.

By design, the overall cost to a university of implementing the compact, in the short term, would be quite small. Hybrid open-access fees are explicitly eschewed, and true open-access fees tend to be found at present in just those areas of scholarship where grant support is most prevalent, reducing the underwriting load on the university substantially. Rough estimates based on the experience of the Berkeley Research Impact Initiative fall in the range of tens of dollars per faculty member per year. In the longer term, as publishers switch journals to an open-access processing-fee model, costs will increase, but these increases will be offset by the compensatory elimination of subscription fees and improvements in efficiency from repairing the market dysfunction that has plagued the subscription-based model, and will be accompanied by a broadening of access to scholarship that is central to the universities' and funding agencies' mission. Similarly, the cost to funding agencies can be managed as well. As costs begin to increase for open-access processing fees paid by a funder, compensatory decreases to grant overhead rates can be made to maintain cost neutrality. Such adjustments are appropriate since the processing fee payments are direct costs that are substituting for indirect costs of library subscriptions.

It is important to keep in mind that the goal of the compact is not to increase access to the individual articles it underwrites. That goal is already reasonably satisfied by the possibility of open-access self-archiving that any author can unilaterally perform and that various open-access policies such as that of the National Institutes of Health promote. Rather, the goal of open-access funds as envisioned in the present proposal is to reduce the disincentives to authors and thus the risk to publishers of the processing-fee business model.

(For this reason, the present proposal, though consistent with the recommendation of Universities UK and the Research Information Network in their report on paying open-access charges [3] that universities establish open-access funds, contraindicates aspects of the operations of such funds, such as payment of hybrid charges, that are also consistent with their guidance. Similarly, a small number of universities, beginning with the University of North Carolina in 2005 and including University of Nottingham, Eidgenössische Technische Hochschule (ETH) Zurich, and University of California at Berkeley, have already established funds to pay processing fees. Such pioneers should be commended. However, most of these funds diverge from the policies proposed here, again especially in their general support for hybrid fees. ETH Zurich is a notable exception.)

If all schools and funders committed to the compact, a publisher could more safely move a journal to an open-access processing-fee business model without fear that authors would desert the journal for pecuniary reasons. Support for the compact would also send a signal to publishers and scholarly societies that research universities and funders appreciate and value their contributions and that universities and funders promoting self-archiving have every intention of continuing to fund publication, albeit within a different model. Publishers willing to take a risk will be met by universities and funding agencies willing to support their bold move.

The new US administration could implement such a system through simple FRPAA-like legislation requiring funding agencies to commit to this open-access compact in a cost-neutral manner. Perhaps reimbursement would be limited to authors at universities and research institutions that themselves commit to a similar compact. As funding agencies and universities take on this commitment, we might transition to an efficient, sustainable journal publishing system in which publishers choose freely among business models on an equal footing, to the benefit of all.
